# The pulsed ultrasound strategy effectively decreases the *S. aureus* population of chronic rhinosinusitis patients

**DOI:** 10.1186/s13104-019-4579-3

**Published:** 2019-09-13

**Authors:** Narjes Feizabadi, Javad Sarrafzadeh, Mojtaba Fathali, Behnoosh Vasaghi-Gharamaleki, Mahdi Dadgoo, Jalil Kardan-Yamchi, Hossein Kazemian, Sonia Hesam-Shariati, Mohammad Mehdi Feizabadi

**Affiliations:** 1 Department of Physiotherapy, Faculty of Rehabilitation, Iran University of Medical Sciences, Hemmat Highway, Tehran, Iran; 2 Department of Physiotherapy, Faculty of Rehabilitation, Tehran University of Medical Sciences, Tehran, Iran; 3 Department of Basic Sciences, Faculty of Rehabilitation, Iran University of Medical Sciences, Tehran, Iran; 4 Department of Medical Microbiology, School of Medicine, Tehran University of Medical Sciences, Poursina Street, Enghelabe-Eslami Avenue, Tehran, Iran; 5 Department of Microbiology, Faculty of Medicine, Ilam University of Medical Sciences, Ilam, Iran

**Keywords:** Pulsed ultrasound, Chronic rhinosinusitis, *Staphylococcus aureus*, Treatment, Real-time PCR

## Abstract

**Objective:**

*Staphylococcus aureus* with the ability of biofilm formation and the drug resistance acquisition is one of the most frequently isolated pathogens from chronic rhinosinusitis patients. Ultrasound as an alternative therapy is effectively able to kill the bacteria by cavitation in or on the bacterial cells and peroxide generation and hence improving antibiotic treatment efficacy.

**Results:**

*Staphylococcus aureus* was detected in 4 and 6 out of 14 patients by phenotypic and qPCR assays, respectively. Four patients were completely resolved after pulsed ultrasound treatment. However, presence of the *S. aureus* was confirmed in three healthy controls by bacterial cultivation. Pulsed ultrasound have been quantitatively decreased the *S. aureus* population in chronic rhinosinusitis patients (p < 0.05). Further studies need to be investigated the effectiveness of pulsed ultrasound as an alternative course of CRS patient’s treatment.

## Introduction

Bacterial communities within a polymeric matrix which called biofilms are difficult for cultivation and highly persistent during antibiotic treatment [1]. Approximately, 65% of human bacterial infections are accompanied with biofilms including sinusitis [2]. Chronic rhinosinusitis (CRS) is a prevalent disease and have been documented with bacterial biofilms complicated to control. *Staphylococcus aureus* with the ability of biofilm formation and the drug resistance acquisition is one of the most frequently isolated pathogens from CRS patients [3]. Because of the complex and not well-known nature of the rhinosinusitis and drug resistance development of involved pathogens, treatment of the disease is sophisticated [4]. Medical management of the CRS is difficult and needs a prolonged antibiotic therapy. In addition to the complications of long-term medical treatment, persistence promotion and emerging the drug resistant bacterial populations made investigators to explore alternative therapies [4, 5].

Therapeutic ultrasound (US) has been advocated as a treatment option for CRS patients in recent years [1, 4]. The high-frequency sound waves are used in ultrasound treatment and can be implemented as continuous or pulsed type. Ultrasound is effectively able to kill the bacteria by cavitation in or on the bacterial cells and peroxide generation and hence improving antibiotic treatment efficacy [1]. It has been demonstrated that ultrasound could completely treat CRS patients [6]. Therefore, the aim of this study was to survey the impact of pulsed ultrasound on the *S. aureus* population of chronic rhinosinusitis patients.

## Main text

### Materials and methods

#### Study setting

This study was a double blind, randomized clinical trial performed from April to September 2018. The included individuals were CRS infected adult patients confirmed by clinical diagnostic criteria (more than or equal to two major symptoms or one major symptom (nasal obstruction, facial pain/pressure, postnasal drip and hyposmia) and two minor symptoms (headache, halitosis, fatigue, dental pain and ear pain)) and also the CT scan results based on the Lund-Mackay staging system [7]. Exclusion criteria were having any head and neck malignancy. Totally, fourteen CRS patients were referred by an Ear, Nose, and Throat (ENT) surgeon. Additionally, ten voluntarily healthy people were enrolled in this study. The informed consent form was filled before entering the study.

#### Specimen collection

Specimen collection from all participants were carried out in two times within a 21 days interval (before and after of US exposure to CRS patients, first and second time without any therapy for healthy control group). Briefly, Dacron swabs was used to collect specimens from three sites, including nasopharynx, the right and left meatus and kept in sterile tubes, containing 1 ml saline solution. Following sampling, all specimens were transferred to clinical laboratory for further analysis. Also, CT scan was done for all precipitants in two times, as described above and SNOT-20 questionnaire (Additional file 1) was filled out.

#### Therapeutic ultrasound

Patients were subjected to 10 times pulsed US therapy with 4 cm/s of the probe velocity movement. Ultrasound frequency was 1 MHz. The intensity for maxillary and frontal sinuses were reported 1 W/cm^2^ and 0.5 W/cm^2^, respectively. Duty cycle for ultrasound was 10%. Specimen collection, CT scans and SNOT20 questionnaire was repeated following the last exposure of pulsed US therapy. If recovery was not seen by CT scan findings or patient didn’t report recovery, routinely medical therapy started for patients by ENT specialist.

#### Phenotypic identification by conventional methods

All the specimens were vortexed carefully and worked out as soon as possible after collection. Ten microliters of each specimen were subjected to serial dilution from 10^1^ to 10^−6^ concentration in PBS. By spreading plate technique, 10 microliters of each diluted suspensions were cultured separately in 2 sheep Blood agar, 2 chocolate agar and MacConkey agar plates and were incubated aerobically and anaerobically at 37 °C for 24 h. After the incubation period, the colony-forming units (CFUs)/ml of each specimen was analyzed using Miles and Misra Method [8]. Also, the Gram staining, standard routine biochemical and microbiological tests were used to identify all the bacterial colonies [9].

#### DNA extraction

Total Bacterial DNA was extracted from clinical specimens using Macherey–Nagel (MN) DNA purification kit according to the manufacturer’s instruction (Macherey–Nagel, Germany). Finally, extracted DNA was stored in − 20 °C for further analysis. *S. aureus* ATCC 29213 strain was used as standard strain.

#### Quantitative real time PCR

The primers and probe were used to detect fibronectin-binding protein A (FnbA) gene of *S. aureus* are as follows: forward primer 5-AGTGAGCGACCATACAACAG-3, reverse primer 5-CATAATTCCCGTGACCATTT-3 and probe 5-FAM-AAGCACAAGGACCAATCGAGG-BHQ-1-3 [10]. First, by using the Nanodrop 1000 (Thermo Scientific, USA), the amounts of DNA templates were checked. For calculating the DNA molecules of each template per gram, following formula was used; molecules of DNA = mass (in gram) Avogadro’s number/average molecular weight of a base × template length [11]. A standard curve was drawn using tenfold serially diluted DNA to gain the efficacy of the PCR reactions. The qPCR assay was performed by LinGene K Real Time PCR apparatus (Bioer, Hangzhou, China). The control strain was *S. aureus* ATCC 29213 and all the samples were run in duplicate.

#### Statistical analysis

All data were analyzed by SPSS software version 21. The presence of *S. aureus* among patients and control groups were analyzed by Chi Square test. *S. aureus* detected in samples before and after the therapy by conventional culture technique were analyzed by nonparametric McNemar’s test. For qPCR results between related groups before and after the treatment, Wilcoxon test was used. A P value of < 0.05 was considered as statistically significant.

### Results

#### Bacterial isolates

According to conventional bacterial identification, *S. aureus* was detected in both CRS patients and healthy group before treatment. In this case, *S. aureus* was detected in 3 and 4 cases in healthy and CRS patients, respectively. Importantly, although *S. aureus* prevalence among CRS patients and healthy group was not significant (p > 0.05), but the presence of other pathogenic bacteria such as *Streptococcus pneumoniae*, *Streptococcus pyogenes*, *Haemophilus influenza*, *Klebsiella pneumoniae* and *Escherichia coli* was detected only in CRS patients.

#### Ultrasound treatment output

**Fig. 1:**
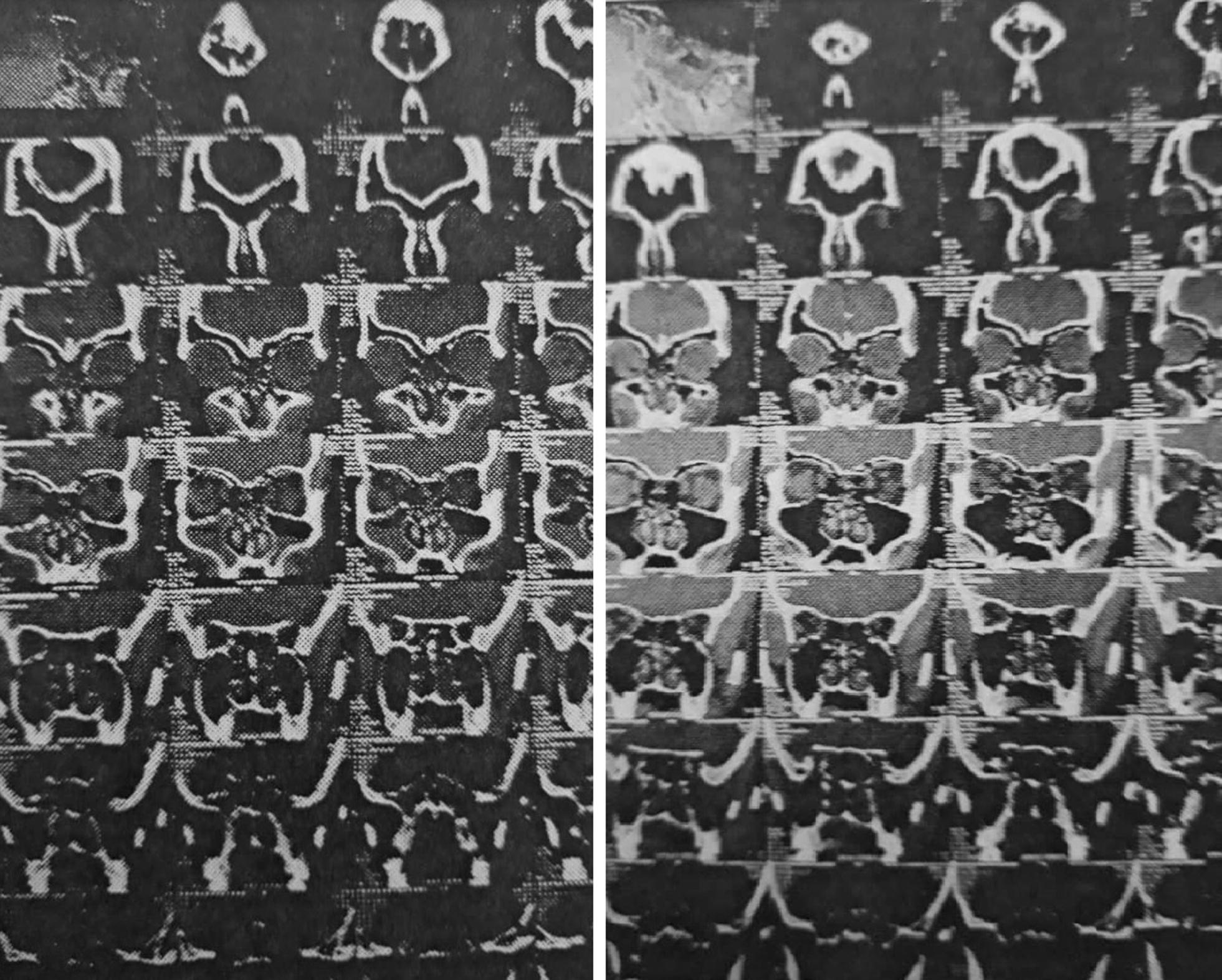
Pre-treatment (left) and post-treatment (right) findings by CT scan

US therapy effectively resolved *S. aureus* in 3 cases and decreased bacterial CFU/ml in one case by phenotypic analysis. This decreased bacterial load was in concomitant with the site changing of *S. aureus* from the right meatus to the nasopharynx. Although this difference is considered to be not statistically significant (p > 0.05), but is clinically significant. In addition, *S. pneumoniae*, *S. pyogenes*, *H. influenza*, *K. pneumoniae* and *E. coli* were removed from different sites of patients except one case that, decreasing of the *Streptococcus pneumoniae* load was seen phenotypically. The CT score of all the patients was decreased considerably after the treatment. As well as, the patient’s scores from the SNOT-20 questionnaire were less after treatment compared to the initial stage. All these findings are in favor of patient’s remission due to pulsed ultrasound treatment (Table 1, Fig. 1).

**Table 1:** The patient’s scores from CT and SNOT-20 questionnaire before and after the treatment

Patient ID	CT scores		Questionnaire scores	
	Before	After	Before	After
1	8	1	56	10
2	17	0	38	8
3	15	5	48	25
4	7	0	24	1
5	27	4	36	9
6	6	1	17	2
7	18	4	17	1
8	24	6	40	25
9	33	11	85	25
10	11	2	45	15
11	6	0	64	1
12	17	2	48	6
13	24	0	54	0
14	17	2	62	10

The CT score and questionnaire scores were decreased after treatment showing patient’s remission

#### Quantitative PCR findings

**Fig. 2:**
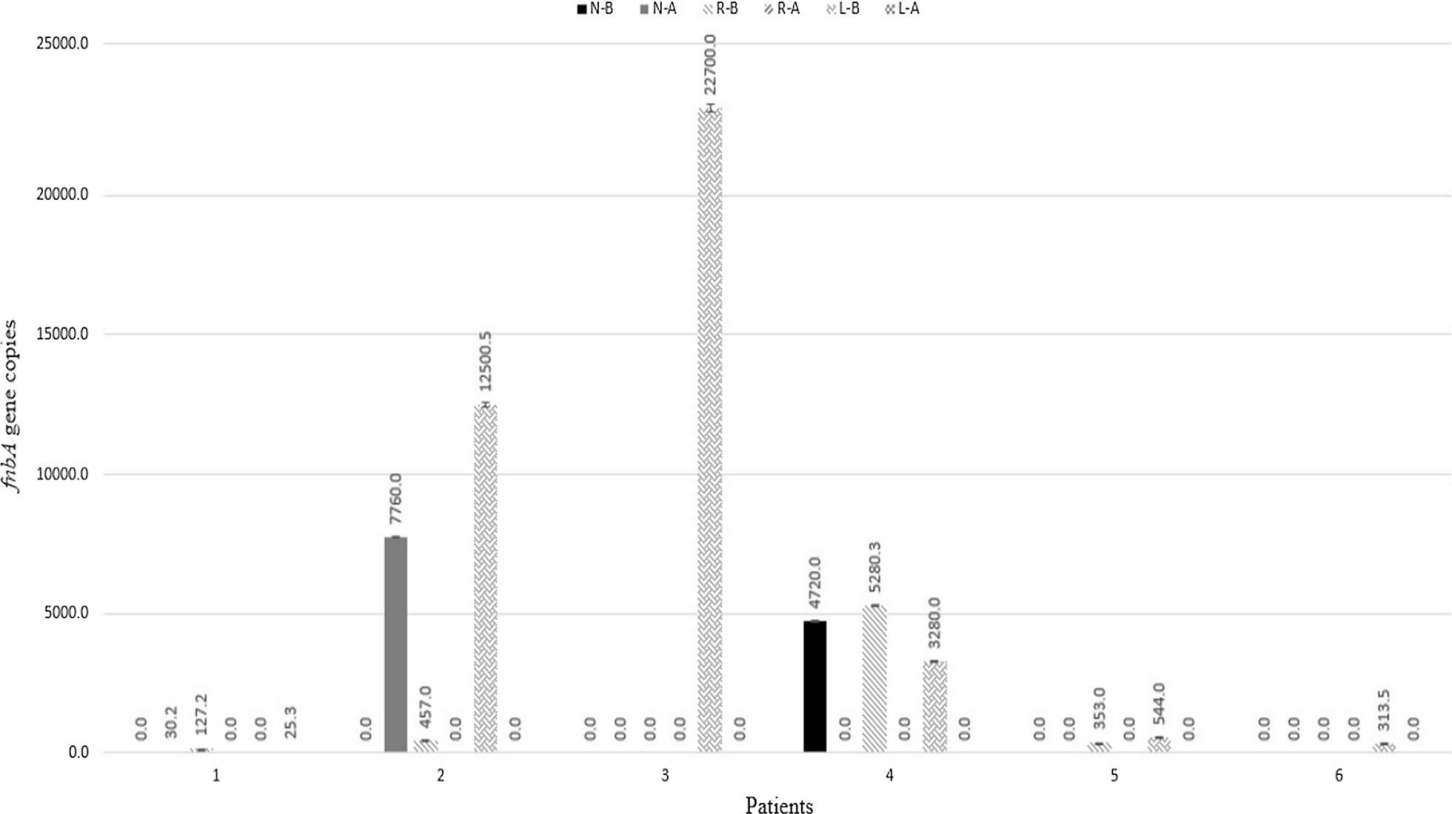
qPCR results of *fnbA* gene in positive patients. (*N-B* nasopharynx-before treatment, *N-A* nasopharynx-after treatment, *R-B* right meatus-before treatment, *R-A* right meatus-after treatment, *L-B* left meatus-before treatment, *L-A* left meatus-after treatment)

Six patients were quantitatively demonstrated to have *S. aureus*. From these, 4 patients were completely recovered after treatment and the bacterial load was significantly reduced (p = 0.02). However, two patients (No. 1, 2) that were negative at the beginning showed *S. aureus* after treatment. This may be because of bacterial transferring during sampling or subsequent colonization of bacteria. The qPCR results are detailed in Fig. 2.

### Discussion

Treatment of CRS patients is encountering to the problem and is challengeable. Mucosal surfaces of the sinuses of these patients is source of multi species of bacterial population within the extracellular matrix which called biofilm [10]. This matrix is elaborated by bacterial population and it causes tolerance and resistance against harsh conditions such as the presence of antimicrobial agents [12]. Already, *S. aureus* is considered as an important infectious agent which can produce the thick layer of biofilm [13, 14].

Despite classical antibiotic therapy in CRS patients commonly is led to failure in therapy, but antibiotics are used as an empiric choice for CRS patients [15, 16] which resulting development of resistant bacteria and thus treatment failure [16]. It has also been shown, exposure of *S. aureus* living in the sinuses to antibiotics led emerging and distributing of methicillin resistant *S. aureus* (MRSA) in CRS patients [16]. Complication of CRS patients by the noticed challenges convinced researchers to study and elaborate alternative strategy for treatment of CRS patients [17]. In this regard, US therapy is a suggested strategy for treatment of CRS patients by breaking down the biofilm structure of the bacterial population. It has been reported that, US can decrease bacterial load through biofilm destruction [18]. In pulsed US strategy, it reduces thermal activities by allowing time for heat to dissipate from the coupling medium during treatment [19]. Although, biofilm formation of bacterial strains and its disruption by therapy was not evaluated in our study, but according to previous studies and published paper {see above), it has been demonstrated that, *S. aureus* is considered as an important CRS agent which can produce the thick layer of biofilm. In another hand, according to literature, US therapy was using as biofilm disruption option [13–18].

It has also been shown, Pulsed US therapy combined with gentamicin decrease bacterial viability in *E. coli* model [1, 20]. A study conducted on pulsed US therapy against *P. aeruginosa*, *E. coli* in implants [21]. These researchers reported that, pulsed US therapy only was effective against *E. coli*. It shows pulsed US therapy is more effective when combined with the antibiotics, which can decrease bacterial viability dramatically [22]. Ansari et al. [23] treated 57 CRS cases using low-intensity pulsed US successfully. According to their reports, most major and minor symptoms indicated significant changes after pulsed US therapy. Young et al. [24] treated 22 CRS cases with six treatments of low-intensity pulsed US which who were being considered for endoscopic sinus surgery. In accordance to these studies, our results derived from bacterial culture and quantitative molecular tests showed a significant reduction in *S. aureus* load in majority of patients after pulsed US treatment.

In conclusion, the clinical improvements of patients showed beneficial recovery and significantly reduced bacterial load were seen in target patients. However, the effectiveness of pulsed ultrasound is thought to be worthy of investigation in further studies.

## Limitations

The low number of volunteers to participate in the study and difficulty to access and following up of all members are limitation of this study.

## Supplementary information

**Additional file 1.** SINO-NASAL OUTCOME TEST (SNOT-20).

## Data Availability

The datasets used and/or analyzed during the current study are presented within the manuscript, supplementary file and also additional information such as patients’ history are available from the corresponding author on reasonable request.

## Abbreviations

CRS: chronic rhinosinusitis
US: ultrasound
CT scan: computed tomography scan
ENT surgeon: nose, and throat surgeon
SNOT: Sino-Nasal Outcome test
CFUs: colony-forming units
FnbA: fibronectin-binding protein A
qPCR: quantitative real time PCR
