# Supplementary material for: The pulsed ultrasound strategy effectively decreases the S. aureus population of chronic rhinosinusitis patients
Source: BMC Res Notes. 2019 Sep 13;12:576. doi: 10.1186/s13104-019-4579-3 (PMC6743179; doi:10.1186/s13104-019-4579-3)
Supplement: Supplementary file 1 — Additional file 1. SINO-NASAL OUTCOME TEST (SNOT-20). [file 13104_2019_4579_MOESM1_ESM.pdf]

I.D.:________________ **SINO-NASAL OUTCOME TEST (SNOT-20)** DATE:________________

Below you will find a list of symptoms and social/emotional consequences of your rhinosinusitis. We would like to know more about these problems and would appreciate your answering the following questions to the best of your ability. There are no right or wrong answers, and only you can provide us with this information. Please rate your problems as they have been over the past two weeks. Thank you for your participation. Do not hesitate to ask for assistance if necessary.

1. Considering how severe the problem is when you experience it and how frequently it happens, please rate each item below on how "bad" it is by circling the number that corresponds with how you feel using this scale: →	No problem	Very mild problem	Mild or slight problem	Moderate Problem	Severe Problem	Problem as bad as it can be	5 Most Important Items
**1. Need to blow nose**	**0**	**1**	**2**	**3**	**4**	**5**	○
**2. Sneezing**	**0**	**1**	**2**	**3**	**4**	**5**	○
**3. Runny nose**	**0**	**1**	**2**	**3**	**4**	**5**	○
**4. Cough**	**0**	**1**	**2**	**3**	**4**	**5**	○
**5. Post-nasal discharge**	**0**	**1**	**2**	**3**	**4**	**5**	○
**6. Thick nasal discharge**	**0**	**1**	**2**	**3**	**4**	**5**	○
**7. Ear fullness**	**0**	**1**	**2**	**3**	**4**	**5**	○
**8. Dizziness**	**0**	**1**	**2**	**3**	**4**	**5**	○
**9. Ear pain**	**0**	**1**	**2**	**3**	**4**	**5**	○
**10. Facial pain/pressure**	**0**	**1**	**2**	**3**	**4**	**5**	○
**11. Difficulty falling asleep**	**0**	**1**	**2**	**3**	**4**	**5**	○
**12. Wake up at night**	**0**	**1**	**2**	**3**	**4**	**5**	○
**13. Lack of a good night's sleep**	**0**	**1**	**2**	**3**	**4**	**5**	○
**14. Wake up tired**	**0**	**1**	**2**	**3**	**4**	**5**	○
**15. Fatigue**	**0**	**1**	**2**	**3**	**4**	**5**	○
**16. Reduced productivity**	**0**	**1**	**2**	**3**	**4**	**5**	○
**17. Reduced concentration**	**0**	**1**	**2**	**3**	**4**	**5**	○
**18. Frustrated/restless/irritable**	**0**	**1**	**2**	**3**	**4**	**5**	○
**19. Sad**	**0**	**1**	**2**	**3**	**4**	**5**	○
**20. Embarrassed**	**0**	**1**	**2**	**3**	**4**	**5**	○

2. Please mark the most important items affecting your health (maximum of 5 items)________________________ ↑

Copyright © 1996 by Jay F. Piccirillo, M.D., Washington University School of Medicine, St. Louis, Missouri
